# Circulation of Influenza A(H5N8) Virus, Saudi Arabia

**DOI:** 10.3201/eid2410.180846

**Published:** 2018-10

**Authors:** Hussain Al-Ghadeer, Daniel K.W. Chu, Ehab M.A. Rihan, Ehab M. Abd-Allah, Haogao Gu, Alex W.H. Chin, Ibrahim A. Qasim, Ali Aldoweriej, Sanad S. Alharbi, Marshad A. Al-Aqil, Ali Al-Sahaf, Salah S. Abdel Rahman, Ali H. Aljassem, Ali Abdul-Al, Mohammed R. Aljasir, Yousef M.O. Alhammad, Samy Kasem, Malik Peiris, Ahmed Z.S.A. Zaki, Leo L.M. Poon

**Affiliations:** Ministry of Environment, Water, and Agriculture, Riyadh, Saudi Arabia (H. Al-Ghadeer, E.M.A. Rihan, E.A. Abd-Allah, I.A. Qasim, A. Aldoweriej, S.S. Alharbi, M.A. Al-Aqil, A. Al-Sahaf, S.S. Abdel Rahman, A.H. Aljassem, A. Abdul-Al, M.R. Aljasir, Y.M.O. Alhammad, S. Kasem, A.Z.S.A. Zaki);; University of Hong Kong, Hong Kong (D.K.W. Chu, H. Gu, A.W.H. Chin, M. Peiris, L.L.M. Poon);; Kafr El-Sheikh University, Kafr El-Sheikh, Egypt (S. Kasem)

**Keywords:** influenza, influenza A(H5N8), outbreak, Saudi Arabia, phylogenetic analysis, viruses

## Abstract

Highly pathogenic avian influenza A(H5N8) viruses have been detected in several continents. However, limited viral sequence data are available from countries in the Middle East. We report full-genome analyses of highly pathogenic H5N8 viruses recently detected in different provinces in Saudi Arabia.

On December 19, 2017, a high number of dead birds from various species was reported in a live bird market in Riyadh, Saudi Arabia, by the Department of Animal Resources Services, Ministry of Environment, Water, and Agriculture. Oropharyngeal and cloacal swab samples were collected from affected birds and investigated for highly pathogenic avian influenza (HPAI) viruses in Riyadh Veterinary Diagnostic Laboratory using reverse transcription PCR (RT-PCR) (*1*). These tests detected HPAI A(H5N8) virus. After this index outbreak, HPAI was reported in adjacent provinces. Surveillance studies were performed in all provinces (>1 major poultry market and 10 backyard farms per province) to estimate disease prevalence. As of May 2018, a total of 7,273 birds had been investigated; 805 were positive for H5N8, which was detected in 7 provinces (Riyadh, Eastern, Al-Qasim, Makkah, Al-Madinah, Asir, and Jizan). The highest number of positive results was reported in Riyadh (693 samples), in which different commercial poultry farms (22 farms for laying hens, 2 for broiler breeders, and 1 for quail) were affected. A contingency plan, based on a stamping-out policy, was implemented to control the disease. More than 8.8 million birds were depopulated.

Positive clinical specimens (N = 14) collected from different settings, different provinces, different avian species or a combination were sent to a World Health Organization H5 reference laboratory in Hong Kong for confirmation. All samples tested positive for membrane protein (M) and hemagglutinin (HA) subtype H5 genes by RT-PCR (Technical Appendix Table 1). Samples that had a cycle threshold value <29 in the M gene assay also tested positive for N8 by RT-PCR. Ten of these samples were positive for virus isolation in embryonated chicken eggs and were associated with death of the chicken embryos by day 3 postinoculation. 

We amplified viral RNA extracted from the clinical specimens and virus isolates using a multisegment RT-PCR approach for full-genome amplification (*2*). We subjected the RT-PCR products to next-generation sequencing on an Illumina MiSeq (PE300) platform (Illumina, San Diego, CA, USA). We edited the deduced consensus sequences (average sequence coverage >10,000×) using BioEdit (https://www.mbio.ncsu.edu/BioEdit/bioedit.html) and analyzed them phylogenetically using MEGA7 (https://www.megasoftware.net*)* (GISAID accession nos. for reference sequences, EPI1215422–EPI1215461, EPI1215137–EPI1215184; http://platform.gisaid.org).

The deduced sequences revealed that H5N8 viruses (n = 11) from different sites in Saudi Arabia are almost identical (sequence identity >99.7%), indicating a common origin for this outbreak. Phylogenetic analyses of HA sequences showed that they belong to clade 2.3.4.4 group B (Figure) (*3*). Polymerase acidic protein (PA), HA, nucleoprotein (NP), neuraminidase (NA), M, and nonstructural protein (NS) segments were genetically similar to those derived from recent group B H5N8 viruses (Technical Appendix Table 2, Figure 1). No genetic markers associated with mammalian host adaptation, α2,6 receptor-binding specificity, or antimicrobial drug resistance were detected (data not shown) (*4*). The gene constellation of PA, HA, NP, NA, M, and NS segments of these H5N8 viruses is similar to those of some H5N8 viruses detected in wild migratory birds from different geographic areas (e.g., A/Anser_cygnoides/Hubei/FW44/2016 and A/green-winged teal/Egypt/877/2016) (*4*,*5*). The polymerase basic protein (PB) 1 and 2 segments of these viruses are similar to those of HPAI H5N5 viruses detected in the Far East (e.g., A/environment/Kamchatka/18/2016) and Europe (e.g., A/swan/Germany-SN/R10645/2016) (Technical Appendix Figure 1). H5N5 viruses of this lineage were previously proposed to be reassortants of an H5N8 virus (*6*), with the PB1 and PB2 segments derived from an H10 virus (A/duck/Mongolia/245/2015-like virus) and the PA, HA, M, and NS segments derived from a H5N8 virus. Our results agree with previous observations that H5N8 viruses of this lineage continue to evolve and reassort with other influenza virus subtypes in migratory bird populations (*7*,*8*).

**Figure:**
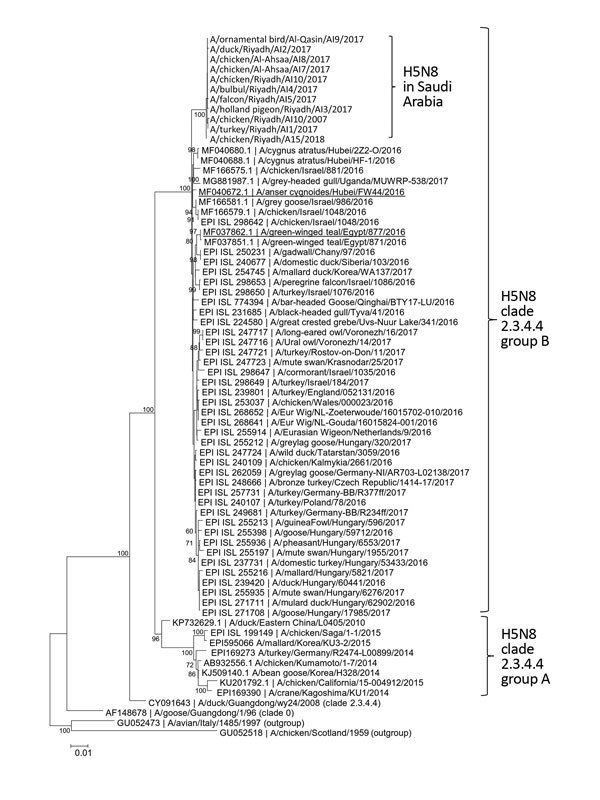
Phylogenetic analysis of hemagglutinin sequences of influenza A(H5N8) viruses detected in oropharyngeal and cloacal swab samples from birds in Saudi Arabia. Aligned sequences were analyzed in MEGA7 (http://www.megasoftware.net). We constructed the phylogenetic tree using the neighbor-joining method. Representative viral sequences and viral sequences that are highly similar to those reported in this study were included in the analysis. H5N8 viruses reported in this study are labeled. Bootstrap values ≥60% are shown. Representative viruses sharing a similar gene constellation as the H5N8 viruses found in Saudi Arabia are underlined (see text for details). Virus isolate numbers (EPI ISL) in GISAID (http://platform.gisaid.org) or gene accession numbers in GenBank for corresponding viral sequences are provided. Scale bar indicates estimated genetic distance.

The studied samples were collected from multiple avian species in different settings from 3 provinces (Technical Appendix Table 1). Of 986 samples from poultry holding sites, 182 (18.5%) tested positive for H5N8 virus. The transmission pathway of H5N8 virus in Saudi Arabia is being investigated. Molecular dating analyses suggest that the most recent common ancestor of these H5N8 viruses emerged in this country in September 2017 (Technical Appendix Figure 2). The potential roles of wild birds, backyard poultry practices, poultry trading, and other human activities in dissemination of these viruses are yet to be determined. However, our results suggest wide circulation of H5N8 viruses caused by a single introduction.

Recently, outbreaks of H5N8 viruses were reported in the Middle East (Israel, Iran, Iraq, and Kuwait) (*1*). However, with the exception of a few HA sequences (n = 12), no other H5N8 viral sequences from this region are available in major sequence databases, which has hampered the investigation of H5N8 viruses in this region. Multiple introductions of H5N8 viruses with different gene constellations have been reported in Egypt (*9*,*10*), but their genetic relationship to H5N8 viruses detected in other countries in the Middle East is not clear. Further surveillance using full-genome analyses is urgently needed to identify major risk factors for HPAI H5N8 viruses in the Middle East.

Technical Appendix Phylogenetic analysis of influenza A(H5N8), Saudi Arabia.
