# Supplementary material for: Circulation of Influenza A(H5N8) Virus, Saudi Arabia
Source: Emerg Infect Dis. 2018 Oct;24(10):1961–4. doi: 10.3201/eid2410.180846 (PMC6154161; doi:10.3201/eid2410.180846)
Supplement: Technical Appendix — Phylogenetic analysis of influenza A(H5N8), Saudi Arabia. [file 18-0846-Techapp-s1.pdf]

Article DOI: https://doi.org/10.3201/eid2410.180846

Circulation of Influenza H5N8 Virus, Saudi Arabia

Technical Appendix

Technical Appendix Table 1. Influenza A(H5N8) samples reported in this study, Saudi Arabia*

Sample	Sampling province	Sampling site	Sampling date	Type of bird	M†	H5†‡	N8§	Virus isolate	NGS
A/Turkey/Riyadh/AI1/2017	Riyadh	Bird market	2017 Dec 21	Poultry	19.6	23.6	18.0	+	+
A/Duck/Riyadh/AI2/2017	Riyadh	Bird market	2017 Dec 21	Poultry	20.7	24.8	19.8	+	+
A/Holland pigeon/Riyadh/AI3/2017	Riyadh	Bird market	2017 Dec 21	Poultry	26.2	30.9	25.5	+	+¶
A/Bulbul/Riyadh/AI4/2017	Riyadh	Bird shop	2017 Dec 21	Kept bird	30.7	34.0	–#	+	+¶
A/Falcon/Riyadh/AI5/2017	Riyadh	Private owner	2017 Dec 21	Kept bird	24.6	28.0	23.1	+	+¶
A/Chicken/Riyadh/AI6/2017	Riyadh	Poultry farm	2017 Dec 21	Poultry	17.4	20.7	15.7	+	+
A/Chicken/Al-Ahsaa/AI7/2017	Eastern Province	Backyard	2017 Dec 26	Poultry	19.6	23.1	18.7	+	+
A/Chicken/Al-Ahsaa/AI8/2017	Eastern Province	Backyard	2017 Dec 26	Poultry	25.2	28.6	23.8	+	+¶
A/Ornamental bird/Al-Qasim/AI9/2017	Al-Qasim	Backyard	2017 Dec 26	Kept bird	25.0	28.7	24.4	–	+
A/Chicken/Riyadh/AI10/2017	Riyadh	Poultry farm	2017 Dec 28	Poultry	13.9	17.6	12.7	+	+
A/Chicken/Eastern Province/AI11/2017	Eastern Province	Backyard	2017 Dec 28	Poultry	32.5	35.5	–	–	–
A/Goose/Eastern Province/AI12/2017	Eastern Province	Backyard	2017 Dec 28	Poultry	29.5	33.1	–	–	–
A/Duck/Eastern Province/AI13/2017	Eastern Province	Backyard	2017 Dec 28	Poultry	29.4	32.8	–	–	–
A/Chicken/Riyadh/A15/2018	Riyadh	Bird shop	2018 Jan 3	Poultry	22.8	26.2	21.0	+	+

*Numbers denote cycle threshold (CT) values. NGS, next-generation sequencing
†Primer and probe sets were modified from WHO RT-PCR protocols for influenza diagnosis (http://www.who.int/influenza/gisrs_laboratory/molecular_diagnosis/en/). M gene: one-step real-time RT-PCR procedures for the detection of influenza A viruses (protocol 2); H5: one-step real-time RT-PCR procedures for the detection of influenza subtypes H5, H57N9, and H9 (protocol 4).
‡The high Ct values found in this assay were caused by primer mismatches at the reverse primer (5'-AATTCCCTTCCAACGGCCTCAAAC-3'; mismatches are underlined).
§The N6 and N8 RT-PCR assays were modified from Hoffman et al. (https://www.nature.com/articles/srep27211).
¶Virus isolates were used as RNA sources for next-generation sequencing.
# – indicates that the assay produced a negative result.

Technical Appendix Table 2. Viral sequences with the highest sequence identity to those from A/Turkey/Riyadh/AI1/2017

Segment*	GISAID accession no.	Virus (subtype)	Sequence identity
PB2	EPI1010642	A/barnacle goose/Netherlands/2/2014 (H3N6)	98.3%
PB1	EPI961474	A/environment/Kamchatka/18/2016 (H5N5)	98.5%
PA	EPI858843	A/painted stork/India/10CA03/2016 (H5N8)	99.4%
HA	EPI909452	A/wild duck/Tatarstan/3059/2016 (H5N8)	98.7%
NA	EPI926614	A/domestic duck/Siberia/103/2016 (H5N8)	99.1%
NA	EPI1159827	A/Cygnus atratus/Hubei/HF-1/2016 (H5N8)	98.4%
M	EPI1010490	A/green-winged teal/Egypt/871/2016 (H5N8)	99.5%
NS	EPI926617	A/domestic duck/Siberia/103/2016 (H5N8)	99.5%

*HA, hemagglutinin; M, membrane protein; NA, neuraminidase; NS, nonstructural protein; PA, polymerase acidic protein; PB, polymerase basic protein

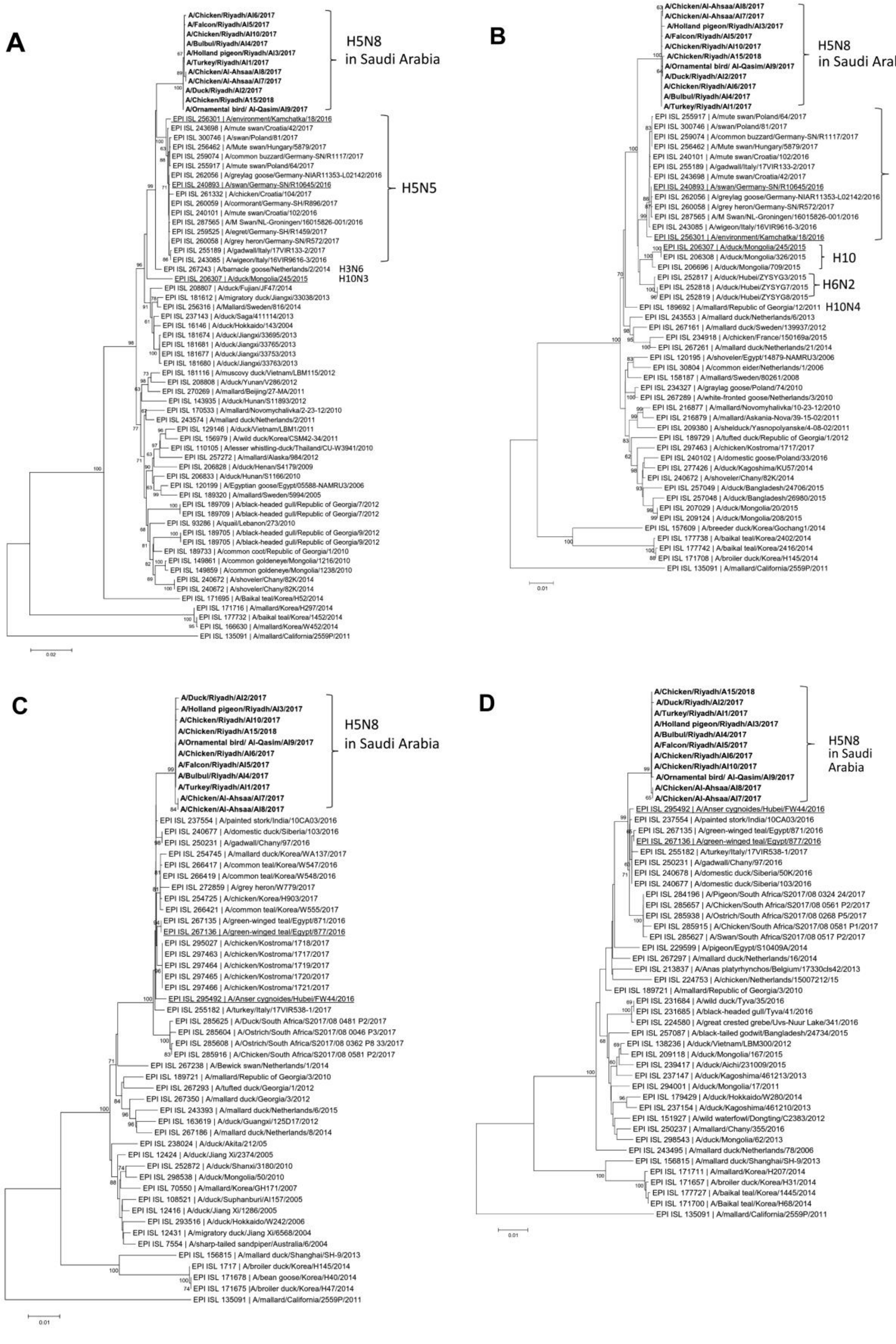
A
H5N8
in Saudi Arabia
H5N5
H3N6
H10N3
B
H5N8
in Saudi Arabia
H5N5
H10
H6N2
H10N4
C
H5N8
in Saudi Arabia
D
H5N8
in Saudi
Arabia

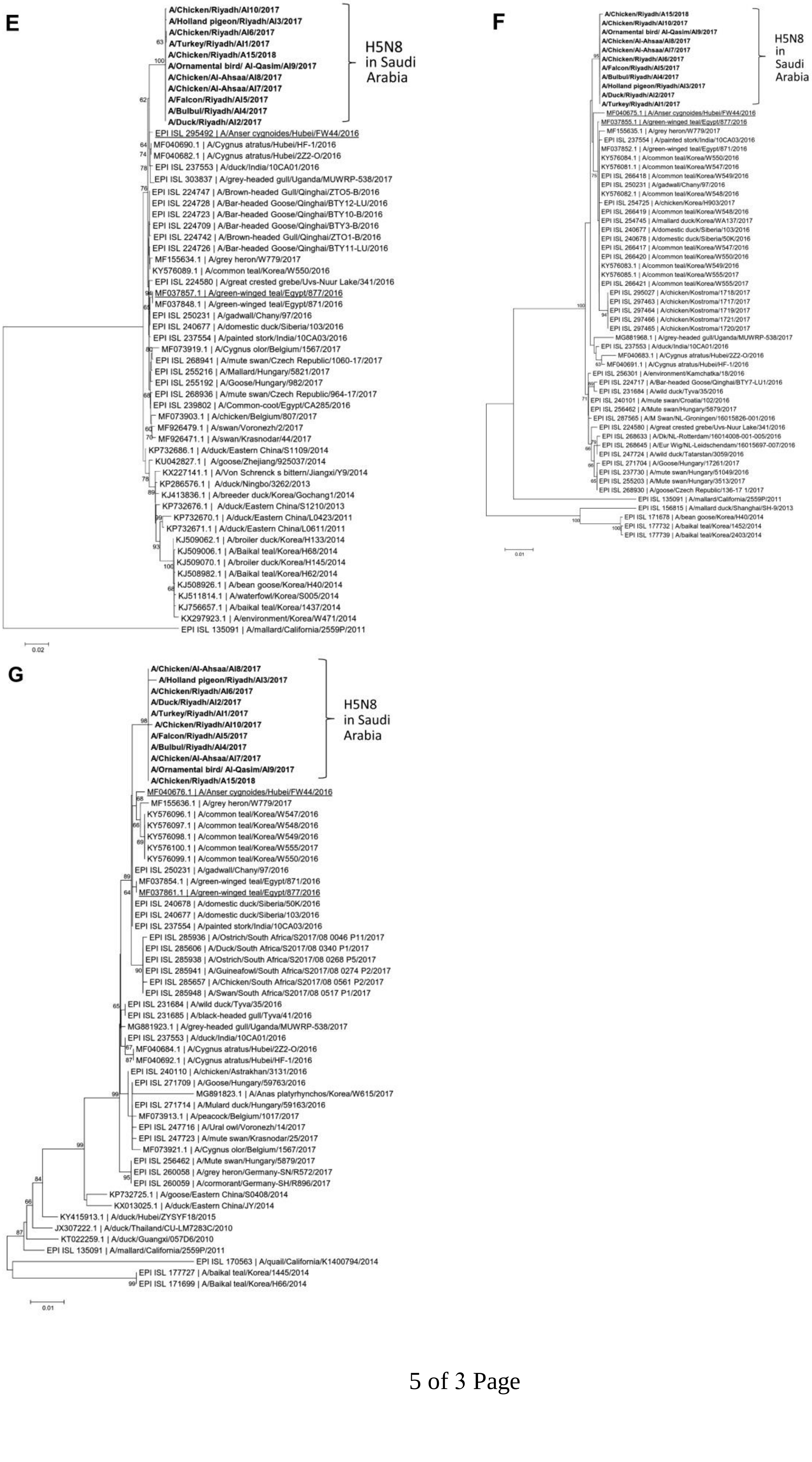
E
A/Chicken/Riyadh/AI10/2017
A/Holland pigeon/Riyadh/AI3/2017
A/Chicken/Riyadh/AI6/2017
A/Turkey/Riyadh/AI1/2017
A/Chicken/Riyadh/A15/2018
A/Ornamental bird/ Al-Qasim/AI9/2017
A/Chicken/Al-Ahsaa/AI8/2017
A/Chicken/Al-Ahsaa/AI7/2017
A/Falcon/Riyadh/AI5/2017
A/Bulbul/Riyadh/AI4/2017
A/Duck/Riyadh/AI2/2017
H5N8 in Saudi Arabia
EPI ISL 295492 | A/Anser cygnoides/Hubei/FW44/2016
MF040690.1 | A/Cygnus atratus/Hubei/HF-1/2016
MF040682.1 | A/Cygnus atratus/Hubei/2Z2-O/2016
EPI ISL 237553 | A/duck/India/10CA01/2016
EPI ISL 303837 | A/grey-headed gull/Uganda/MUWRP-538/2017
EPI ISL 224747 | A/Brown-headed Gull/Qinghai/ZTO5-B/2016
EPI ISL 224728 | A/Bar-headed Goose/Qinghai/BTY12-LU/2016
EPI ISL 224723 | A/Bar-headed Goose/Qinghai/BTY10-B/2016
EPI ISL 224709 | A/Bar-headed Goose/Qinghai/BTY3-B/2016
EPI ISL 224742 | A/Brown-headed Gull/Qinghai/ZTO1-B/2016
EPI ISL 224726 | A/Bar-headed Goose/Qinghai/BTY11-LU/2016
MF155634.1 | A/grey heron/W779/2017
KY576089.1 | A/common teal/Korea/W550/2016
EPI ISL 224580 | A/great crested grebe/Uvs-Nuur Lake/341/2016
MF037857.1 | A/green-winged teal/Egypt/877/2016
MF037848.1 | A/green-winged teal/Egypt/871/2016
EPI ISL 250231 | A/gadwall/Chany/97/2016
EPI ISL 240677 | A/domestic duck/Siberia/103/2016
EPI ISL 237554 | A/painted stork/India/10CA03/2016
MF073919.1 | A/Cygnus olor/Belgium/1567/2017
EPI ISL 268941 | A/mute swan/Czech Republic/1060-17/2017
EPI ISL 255216 | A/Mallard/Hungary/5821/2017
EPI ISL 255192 | A/Goose/Hungary/982/2017
EPI ISL 268936 | A/mute swan/Czech Republic/964-17/2017
EPI ISL 239802 | A/Common-coot/Egypt/CA285/2016
MF073903.1 | A/chicken/Belgium/807/2017
MF926479.1 | A/swan/Voronezh/2/2017
MF926471.1 | A/swan/Krasnodar/44/2017
KP732686.1 | A/duck/Eastern China/S1109/2014
KU042827.1 | A/goose/Zhejiang/925037/2014
KX227141.1 | A/Von Schrenck s bittern/Jiangxi/Y9/2014
KP286576.1 | A/duck/Ningbo/3262/2013
KJ413836.1 | A/breeder duck/Korea/Gochang1/2014
KP732676.1 | A/duck/Eastern China/S1210/2013
KP732670.1 | A/duck/Eastern China/L0423/2011
KP732671.1 | A/duck/Eastern China/L0611/2011
KJ509062.1 | A/broiler duck/Korea/H133/2014
KJ509006.1 | A/Baikal teal/Korea/H68/2014
KJ509070.1 | A/broiler duck/Korea/H145/2014
KJ508982.1 | A/Baikal teal/Korea/H62/2014
KJ508926.1 | A/bean goose/Korea/H40/2014
KJ511814.1 | A/waterfowl/Korea/S005/2014
KJ756657.1 | A/baikal teal/Korea/1437/2014
KX297923.1 | A/environment/Korea/W471/2014
EPI ISL 135091 | A/mallard/California/2559P/2011
0.02
F
A/Chicken/Riyadh/A15/2018
A/Chicken/Riyadh/AI10/2017
A/Ornamental bird/ Al-Qasim/AI9/2017
A/Chicken/Al-Ahsaa/AI8/2017
A/Chicken/Al-Ahsaa/AI7/2017
A/Chicken/Riyadh/AI6/2017
A/Falcon/Riyadh/AI5/2017
A/Bulbul/Riyadh/AI4/2017
A/Holland pigeon/Riyadh/AI3/2017
A/Duck/Riyadh/AI2/2017
A/Turkey/Riyadh/AI1/2017
H5N8 in Saudi Arabia
MF040675.1 | A/Anser cygnoides/Hubei/FW44/2016
MF037855.1 | A/green-winged teal/Egypt/877/2016
MF155635.1 | A/grey heron/W779/2017
EPI ISL 237554 | A/painted stork/India/10CA03/2016
MF037852.1 | A/green-winged teal/Egypt/871/2016
KY576084.1 | A/common teal/Korea/W550/2016
KY576081.1 | A/common teal/Korea/W547/2016
EPI ISL 266418 | A/common teal/Korea/W549/2016
EPI ISL 250231 | A/gadwall/Chany/97/2016
KY576082.1 | A/common teal/Korea/W548/2016
EPI ISL 254725 | A/chicken/Korea/H903/2017
EPI ISL 266419 | A/common teal/Korea/W548/2016
EPI ISL 254745 | A/mallard duck/Korea/WA137/2017
EPI ISL 240677 | A/domestic duck/Siberia/103/2016
EPI ISL 240678 | A/domestic duck/Siberia/50K/2016
EPI ISL 266417 | A/common teal/Korea/W547/2016
EPI ISL 266420 | A/common teal/Korea/W550/2016
KY576083.1 | A/common teal/Korea/W549/2016
KY576085.1 | A/common teal/Korea/W555/2017
EPI ISL 266421 | A/common teal/Korea/W555/2017
EPI ISL 295027 | A/chicken/Kostroma/1718/2017
EPI ISL 297463 | A/chicken/Kostroma/1717/2017
EPI ISL 297464 | A/chicken/Kostroma/1719/2017
EPI ISL 297466 | A/chicken/Kostroma/1721/2017
EPI ISL 297465 | A/chicken/Kostroma/1720/2017
MG881968.1 | A/grey-headed gull/Uganda/MUWRP-538/2017
EPI ISL 237553 | A/duck/India/10CA01/2016
MF040683.1 | A/Cygnus atratus/Hubei/2Z2-O/2016
MF040691.1 | A/Cygnus atratus/Hubei/HF-1/2016
EPI ISL 256301 | A/environment/Kamchatka/18/2016
EPI ISL 224717 | A/Bar-headed Goose/Qinghai/BTY7-LU1/2016
EPI ISL 231684 | A/wild duck/Tyva/35/2016
EPI ISL 240101 | A/mute swan/Croatia/102/2016
EPI ISL 256462 | A/Mute swan/Hungary/5879/2017
EPI ISL 287565 | A/M Swan/NL-Groningen/16015826-001/2016
EPI ISL 224580 | A/great crested grebe/Uvs-Nuur Lake/341/2016
EPI ISL 268633 | A/Dk/NL-Rotterdam/16014008-001-005/2016
EPI ISL 268645 | A/Eur Wig/NL-Leidschendam/16015697-007/2016
EPI ISL 247724 | A/wild duck/Tatarstan/3059/2016
EPI ISL 271704 | A/Goose/Hungary/17261/2017
EPI ISL 237730 | A/mute swan/Hungary/51049/2016
EPI ISL 255203 | A/Mute swan/Hungary/3513/2017
EPI ISL 268930 | A/goose/Czech Republic/136-17 1/2017
EPI ISL 135091 | A/mallard/California/2559P/2011
EPI ISL 156815 | A/mallard duck/Shanghai/SH-9/2013
EPI ISL 171678 | A/bean goose/Korea/H40/2014
EPI ISL 177732 | A/baikal teal/Korea/1452/2014
EPI ISL 177739 | A/baikal teal/Korea/2403/2014
0.01
G
A/Chicken/Al-Ahsaa/AI8/2017
A/Holland pigeon/Riyadh/AI3/2017
A/Chicken/Riyadh/AI6/2017
A/Duck/Riyadh/AI2/2017
A/Turkey/Riyadh/AI1/2017
A/Chicken/Riyadh/AI10/2017
A/Falcon/Riyadh/AI5/2017
A/Bulbul/Riyadh/AI4/2017
A/Chicken/Al-Ahsaa/AI7/2017
A/Ornamental bird/ Al-Qasim/AI9/2017
A/Chicken/Riyadh/A15/2018
H5N8 in Saudi Arabia
MF040676.1 | A/Anser cygnoides/Hubei/FW44/2016
MF155636.1 | A/grey heron/W779/2017
KY576096.1 | A/common teal/Korea/W547/2016
KY576097.1 | A/common teal/Korea/W548/2016
KY576098.1 | A/common teal/Korea/W549/2016
KY576100.1 | A/common teal/Korea/W555/2017
KY576099.1 | A/common teal/Korea/W550/2016
EPI ISL 250231 | A/gadwall/Chany/97/2016
MF037854.1 | A/green-winged teal/Egypt/871/2016
MF037861.1 | A/green-winged teal/Egypt/877/2016
EPI ISL 240678 | A/domestic duck/Siberia/50K/2016
EPI ISL 240677 | A/domestic duck/Siberia/103/2016
EPI ISL 237554 | A/painted stork/India/10CA03/2016
EPI ISL 285936 | A/Ostrich/South Africa/S2017/08 0046 P11/2017
EPI ISL 285606 | A/Duck/South Africa/S2017/08 0340 P1/2017
EPI ISL 285938 | A/Ostrich/South Africa/S2017/08 0268 P5/2017
EPI ISL 285941 | A/Guineafowl/South Africa/S2017/08 0274 P2/2017
EPI ISL 285657 | A/Chicken/South Africa/S2017/08 0561 P2/2017
EPI ISL 285948 | A/Swan/South Africa/S2017/08 0517 P1/2017
EPI ISL 231684 | A/wild duck/Tyva/35/2016
EPI ISL 231685 | A/black-headed gull/Tyva/41/2016
MG881923.1 | A/grey-headed gull/Uganda/MUWRP-538/2017
EPI ISL 237553 | A/duck/India/10CA01/2016
MF040684.1 | A/Cygnus atratus/Hubei/2Z2-O/2016
MF040692.1 | A/Cygnus atratus/Hubei/HF-1/2016
EPI ISL 240110 | A/chicken/Astrakhan/3131/2016
EPI ISL 271709 | A/Goose/Hungary/59763/2016
MG891823.1 | A/Anas platyrhynchos/Korea/W615/2017
EPI ISL 271714 | A/Mulard duck/Hungary/59163/2016
MF073913.1 | A/peacock/Belgium/1017/2017
EPI ISL 247716 | A/Ural owl/Voronezh/14/2017
EPI ISL 247723 | A/mute swan/Krasnodar/25/2017
MF073921.1 | A/Cygnus olor/Belgium/1567/2017
EPI ISL 256462 | A/Mute swan/Hungary/5879/2017
EPI ISL 260058 | A/grey heron/Germany-SN/R572/2017
EPI ISL 260059 | A/cormorant/Germany-SH/R896/2017
KP732725.1 | A/goose/Eastern China/S0408/2014
KX013025.1 | A/duck/Eastern China/JY/2014
KY415913.1 | A/duck/Hubei/ZYSYF18/2015
JX307222.1 | A/duck/Thailand/CU-LM7283C/2010
KT022259.1 | A/duck/Guangxi/057D6/2010
EPI ISL 135091 | A/mallard/California/2559P/2011
EPI ISL 170563 | A/quail/California/K1400794/2014
EPI ISL 177727 | A/baikal teal/Korea/1445/2014
EPI ISL 171699 | A/Baikal teal/Korea/H66/2014
0.01

Technical Appendix Figure 1. Phylogenetic analyses of H5N8 viruses detected in Saudi Arabia: A) polymerase basic protein 2 (PB2); B) PB1; C) polymerase acidic protein (PA); D) nucleoprotein (NP); E) neuraminidase (NA); F) membrane protein (M); G) nonstructural protein (NS). Aligned sequences were analysed by MEGA7 (https://www.megasoftware.net/). Phylogenetic trees were constructed using the neighbor-joining method. Representative viral sequences and viral sequences that are highly similar to those reported in this study were included in these analyses. H5N8 viruses reported in this study are highlighted as shown. Bootstrap values ≥60% are shown. Representative viruses that share a similar gene constellation (PB2 and PB1; PA, HA, NP, NA, M, and NS) of H5N8 viruses found in Saudi Arabia are underlined (see main text for details). GISAID accession numbers for corresponding viral sequences are shown as indicated. Scale bar indicates the estimated genetic distance of these viruses.

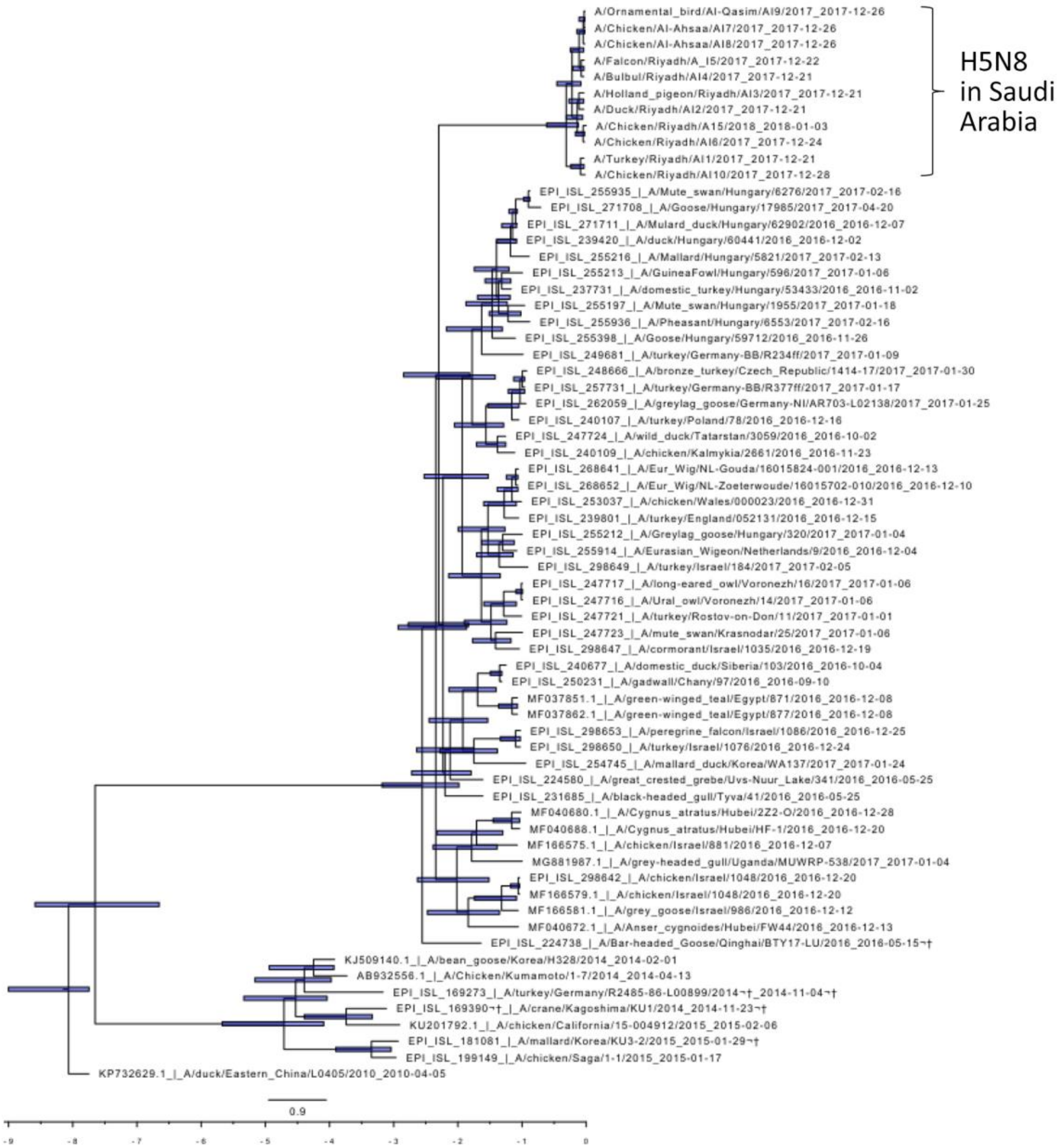

Technical Appendix Figure 2. Phylogenetic tree of hemagglutinin (HA) sequences with dating estimated by BEAST (http://beast.community/). Median (in years) and the estimated posterior probabilities of nodes are shown. Node bars indicate 95% highest posterior density regions of node dating. The median date of the most recent common ancestor of H5N8 viruses in Saudi Arabia is estimated to be September 11, 2017 (95% CI May 23–November 20, 2017). GISAID accession numbers of the reference sequences are indicated.
